# Tinfoil Matrices

**Published:** 1898-10

**Authors:** W. Booth Pearson

**Affiliations:** Dublin, Ireland.


ARTICLE III.

### TINFOIL MATRICES.

W. BOOTH PEARSON, F. R. C. S., DUBLIN, IRELAND.

Oxy-phosphate fillings are a necessity in daily practice, and, as it is not always advisible to use the rubber dam in the case of irritable or very young patients, some other means of excluding moisture must be sought. During the past ten years I have been able to keep such fillings damp proof, during the progress of the chemical union that causes consolidation of the filling in the tooth cavity, by the aid of pieces of tinfoil. The cavity in the tooth is prepared in a suitable manner, and the margins carefully shaped. The tooth cavity is filled with absorbent cotton carefully packed into it, after the use of the warm-air syringe. A piece of tinfoil of suitable thickness is cut out, measuring one inch or one inch and one-half square, and placed in readiness. The filling is carefully mixed into a pasty mass. The cotton wall is removed, and the plastic filling gently packed into every part of the cavity. Some of the overplus of the filling left on the mixing slab is placed on the piece of tin foil, in a suitable position. The tinfoil is quickly placed over the tooth and the cavity, and folded over the lingual and buccal surface as the case may be. The tinfoil is gently brought into position with the forefinger and the thumb, or with the forefingers of the right and left hand. A stroking action of a righthand finger, while the left index finger holds the tinfoil in position within or without the dental arch, will enable the filling to be solidly pressed into place in the cavity of the teeth. The excess of material is thus squeezed over the teeth and the tinfoil can then be gently burnished over the filling so as to make a contour suitable to the case. In the case of bicuspids and molars, the patient can supplement the procedures of the dentist by steadily closing the bite on the tinfoil, and thus give the dentist a true contour of the masticating surface. In many cases where the approximal cavities in maxillary incisors have to be filled, the tinfoil can be folded double, and passed between the teeth before the filling is placed in the cavity. This flexible band of metal can be gently drawn into place round the tooth, and thus cemented on the enamel surface on each side of the cavity, so that the moisture is completely excluded during the setting of the filling. Once the filling sets hard, the tinfoil can be peeled or scraped off the tooth or teeth, and the dentist will find if these details have been skillfully and gently carried out, a well contoured filling with a smooth surface that needs very little final trimming. Sometimes I place a band of tinfoil round a tooth and then pack in the oxy-phosphate filling. The band is brought to its intended place, and I then envelope tooth and band with a square piece of tinfoil smeared with the filling. This is rubbed or folded into shape and position, and all moisture is excluded. With young children tinfoil used in this way will be found of great service to the dentist, as it does not give them any pain or discomfort, while at the same time the filling is kept protected from the action of the saliva during the progress of setting.

I am of opinion that oxy phosphate fillings treated in this way will be found to be harder and more durable under ordinary conditious than when the plastic mass is packed and burnished into position and contoured with oiled instruments. I have had excellent results during the past three years by using both Dr. W. N. Ames' "metalloid" and "oxy-phosphate of copper" fillings in young mouths. I have kept careful records of these fillings by means of charts, so that what I bring forward is something more accurate and valuable than an "opinion" or an "impression" on the matter, or the fact that they have "an unprecedented sale."

Few young children seem to me to bear with patience the use of the rubber dam, and as a willing patient should be the rule rather than the exception with patients of ten years, I offer my experience in the hope that it will meet a want in ordinary practice that is not altogether filled, by the use of rubber dam napkins, absorbent paper, or even saliva ejector. The tinfoil commonly used for filling teeth is much too thin for this purpose; I prefer to use tinfoil somewhat of the stoutness of writing paper, as it will bear the necessary pressure and manipulation without breaking. Sometimes I fold the square of tinfoil into a strip or band of three or four thicknesses, as may suit the space between the teeth, and thus gain sufficient support to bring pressure to make a contour filling above, or below, the gum, as the case may be, in the maxilla or mandible. A few experiments on some natural teeth set in plaster will show any one who cares to carry out the procedure that tinfoil can be made a valuable adjunct in many cases where oxy-phosphate plastic fillings have to be used. I showed this method to several dental friends at my house last August, and they were much pleased with the adaptability of the tinfoil to the purposes I have endeavored to describe.—*Dominion Dental Journal.*

